# Fine Resolution Air Quality Monitoring from a Small Satellite: CHRIS/PROBA

**DOI:** 10.3390/s8127581

**Published:** 2008-11-27

**Authors:** Janet E. Nichol, Man Sing Wong, Yuk Ying Chan

**Affiliations:** Department of Land Surveying and Geo-Informatics, The Hong Kong Polytechnic University, Hong Kong

**Keywords:** Aerosol, surface reflectance, CHRIS/PROBA

## Abstract

Current remote sensing techniques fail to address the task of air quality monitoring over complex regions where multiple pollution sources produce high spatial variability. This is due to a lack of suitable satellite-sensor combinations and appropriate aerosol optical thickness (AOT) retrieval algorithms. The new generation of small satellites, with their lower costs and greater flexibility has the potential to address this problem, with customised platform-sensor combinations dedicated to monitoring single complex regions or mega-cities. This paper demonstrates the ability of the European Space Agency's small satellite sensor CHRIS/PROBA to provide reliable AOT estimates at a spatially detailed level over Hong Kong, using a modified version of the dense dark vegetation (DDV) algorithm devised for MODIS. Since CHRIS has no middle-IR band such as the MODIS 2,100 nm band which is transparent to fine aerosols, the longest waveband of CHRIS, the 1,019 nm band was used to approximate surface reflectance, by the subtraction of an offset derived from synchronous field reflectance spectra. Aerosol reflectance in the blue and red bands was then obtained from the strong empirical relationship observed between the CHRIS 1,019 nm, and the blue and red bands respectively. AOT retrievals for three different dates were shown to be reliable, when compared with AERONET and Microtops II sunphotometers, and a Lidar, as well as air quality data at ground stations. The AOT images exhibited considerable spatial variability over the 11 × 11km image area and were able to indicate both local and long distance sources.

## 1. Introduction

Small satellites have the advantages of lower costs as well as programmable positioning and sensor modes, and can thus be customized to address environmental monitoring tasks which are challenging for routine commercial satellites. Monitoring of aerosol concentrations as an indicator of air quality at local scale is such a problem due to the need for high temporal, spatial and spectral resolution combined in one sensor. This paper demonstrates that a small satellite CHRIS/PROBA has both the spectral and spatial sensitivity to accurately retrieve aerosols at a detailed level, although its orbit does not offer adequate temporal resolution for continuous monitoring.

There is currently no reliable method for the monitoring of air quality over urban areas using remote sensing. Methodologies by Tanré *et al.* [1], Sifakis *et al.* [2], Kaufman and Tanré [3], Hsu *et al.* [4] do not provide consistent results over spatially complex regions due to inadequate spatial and temporal resolution combined with a lack of suitable algorithms. Furthermore there has been little effort to map air quality at detailed level. According to Li *et al.* [5], aerosols over a 50 km^2^ domain do not vary much, except over regions near major emission sources, and most previous remote sensing studies have not addressed variability in air quality at fine resolution. Thus the MODIS standard Aerosol product MOD04 is at the coarse resolution of 10 km. Hsu *et al.'s* [4] deep blue algorithm, which requires two blue bands (eg. MODIS 412 nm and 470 nm bands) for AOT retrieval over bright surfaces has only been demonstrated successfully for large homogeneous surfaces such as deserts, but not for areas of complex land cover. The differential texture analysis method of Sifakis *et al.* [6] and Retalis *et al.* [7] operates at coarse resolutions of ca. 500 m due to the need for a large kernel size for texture analysis. The method has not been validated empirically due to the lack of AOT validation data, and it suffers from land cover changes over time which is common in human-dominated landscapes. In Hong Kong, air quality modeling by the Environmental Protection Department (EPD) suffers from the distant location and uncertainty of the data sources outside Hong Kong, making model output at resolutions higher than 1.5 km meaningless. Even at this coarse resolution very little variation in air quality over the 1,060 km^2^ of Hong Kong's territory is evident, although data from the 14 ground stations suggests substantial spatial variation [8].

This paper demonstrates that a small hyperspectral and programmable satellite sensor – the Compact High Resolution Imaging Spectrometer (CHRIS)/PROBA, which has wavebands sensitive to aerosols in the visible region, can be used for aerosol retrieval, and suggests that such small satellites would be useful for continuous monitoring at detailed level over complex landscapes, given customized orbital parameters. It modifies Kaufman and Tanré's [3] dense dark vegetation (DDV) algorithm for use with CHRIS imagery over a rugged, forested terrain surrounding several urban areas in Hong Kong.

Data collected by the Hong Kong Polytechnic University AERONET station since its establishment in 2005, show aerosol levels to be high, compared with other urban stations worldwide, with, for example a mean AOT of 0.69 for the 440 nm band, compared with 0.57 for Beijing, 0.55 for Singapore, 0.22 for Rome, and 0.24 for Goddard Space Flight Center. It is likely that the largest proportion of Hong Kong's pollution originates from adjacent rapidly industrializing areas of the Chinese mainland, but with only 14 air quality stations, the occurrence and intensity of trans-boundary air pollution is difficult to establish. For example, although Lo *et al.* [9] emphasize the importance of cross-boundary air pollution from the Chinese mainland, and Yuan *et al.* [10] affirm that the source of 60-70% of PM10 is outside Hong Kong, Civic Exchange [11] maintains that local sources are dominant 53% of the time.

The DDV algorithm, was devised for MODIS wavebands to measure the transparency of the atmosphere over areas of dense vegetation which are dark in blue and red bands. The technique compares a shortwave infra-red (SWIR 2,100 nm) band which is almost transparent to fine mode aerosols and thus represents reflectance at the surface (L_surf_), with another such as blue or red which have low reflectance over dense vegetation but are not transparent to aerosols. The reflectance due to aerosol can be obtained from an empirical ratio established between L_surf_ of these long and short wavelength bands. This ratio is given as 0.25 and 0.5 for blue and red bands respectively [12], but different relationships have been established for different environments, and Levy *et al.* [13] found 0.33 and 0.65 over the East Coast USA, whereas Lee *et al.* [14] found 0.31 and 0.88 in Korea. Aerosol reflectance is then converted to a unitless measure, Aerosol Optical Thickness (AOT) using a radiative transfer model such as 6S or SBDART, with the input of other parameters such as atmospheric humidity and aerosol type. A strong empirical relationship between L_surf_ in the SWIR and visible bands is essential for operation of the DDV algorithm, which Remer *et al.* [15] give as R=0.75 and R=0.93 for blue and red bands respectively, but note considerable seasonal variation, as well as between urban and rural, and tropical and non-tropical sites.

## 2. Images

**Figure 1.**
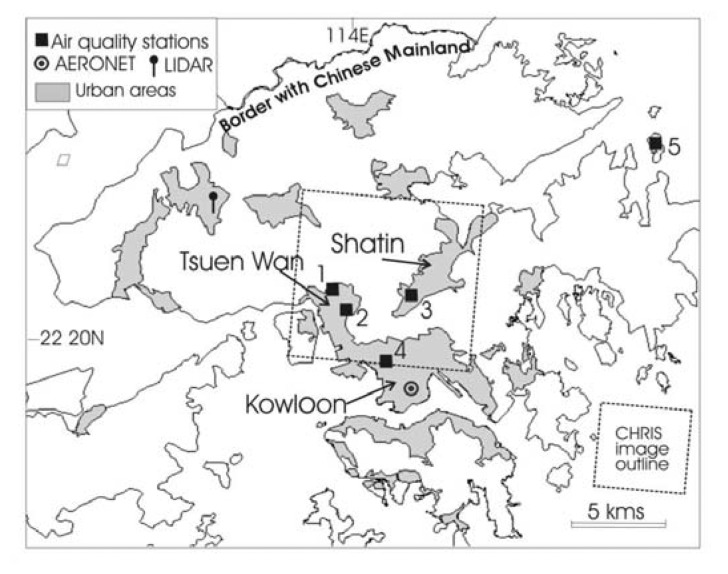
Urban areas in Hong Kong in relation to the area covered by the CHRIS image on 27-Sep-2006, and locations of AERONET, Lidar, and four urban air quality stations: 1. Tsuen Wan, 2. Kwai Chung, 3. Sham Shui Po, 4. Shatin, and 5. Tap Mun (rural station).

The study area for acquisition of the CHRIS/PROBA image was selected to cover both urban and rural areas including the northern part of the urbanized Kowloon Peninsula and the central section of Hong Kong's forested New Territories (Figure 1), although AOT retrieval was carried out only over sites with vegetation.

The Compact High Resolution Imaging Spectrometer (CHRIS) sensor is carried by the Proba satellite which is the first European Space Agency small satellite built for small scientific missions, and is classified as a microsatellite with a mass of 94kg and size of 80cm × 60cm × 60cm. The main scientific objectives of CHRIS are to measure the spectral bidirectional reflectance distribution function (BRDF) for mapping vegetation cover and studying the atmosphere and water bodies. CHRIS is a hyperspectral sensor with five viewing angles, and offers medium spatial, and high spectral and radiometric resolution.

Although its positioning and sensor manouvreability are programmable, its commitment to worldwide users [16] determines its orbit and low repeat cycle, which are unsuitable for continuous monitoring applications. Recent research has demonstrated potential geometric accuracy almost comparable to commercial sensors in spite of the lack of georeferencing information provided [17], and has provided algorithms for effective noise removal [18] and atmospheric correction [19]. However, its radiometric calibration is not ensured [19] and some underestimation of the signal in the NIR region has been noted. The CHRIS image mode 3 (land mode) used for this study offers 18 narrow (mainly 10 nm) VNIR wavebands from 430-1,019 nm at 18 m spatial resolution and 12 bit radiometric resolution. Only nadir images were used in this first phase of the study because of the complexity of estimating surface reflectance for differential illumination conditions in Hong Kong's mountainous terrain, combined with different viewing angles. Several cloud-free images were acquired over Hong Kong for the project, including 18-Dec-2005 and 7-Feb-2006 and 27-Sep-2006. Since CHRIS has no middle infra-red band equivalent to the MODIS 2,100 nm band which is almost transparent to aerosols, the longest waveband of CHRIS, at 1,019 nm, was used for estimating L_surf_. The relationship of the blue and red bands to the 1,019 nm band in this study was found to be slightly higher than between the atmospherically corrected blue and red bands of CHRIS against the SWIR band of Landsat, when this was tested in Hong Kong. The bands used for aerosol estimation were bands 2 (blue at 490 nm), and 7 (red at 661 nm).

## 3. Methodology

### 3.1. Image pre-processing

The images were orthorectified and radiance-to-reflectance converted, to give Top-of Atmosphere reflectance (TOA). Secondly, areas of dense dark vegetation were isolated using the Atmospherically Resistant Vegetation Index (ARVI) [20] and NIR band (Equation 1):

(1) $$\text{if}\;{\text{i}}_{1}<0.8\;\text{or}\;{\text{i}}_{1}>1.6\;\text{or}\;{\text{i}}_{2}<0.1\;\text{then null else}\;{\text{i}}_{3}$$

where i_1_ = ARVI, i_2_ = NIR (1019nm), i_3_ = target image

### 3.2. Estimation of Surface Reflectance (L_surf_)

**Figure 2.**
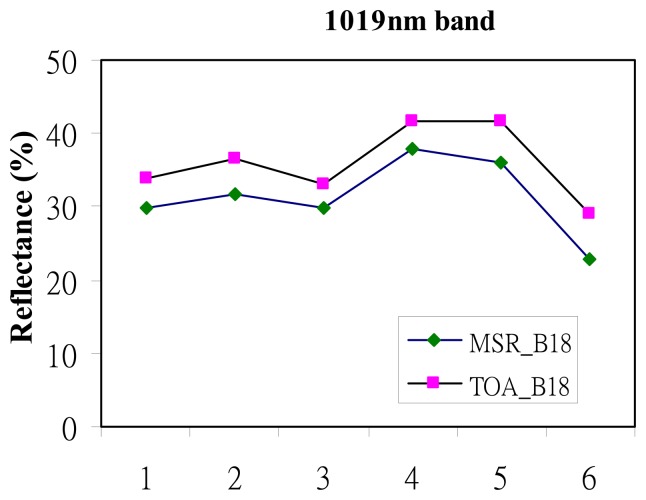
Comparison of MSR reflectance at 6 forest ground sites with TOA reflectance for CHRIS 1,019 nm band. Mean difference is 3.3%.

**Figure 3.**
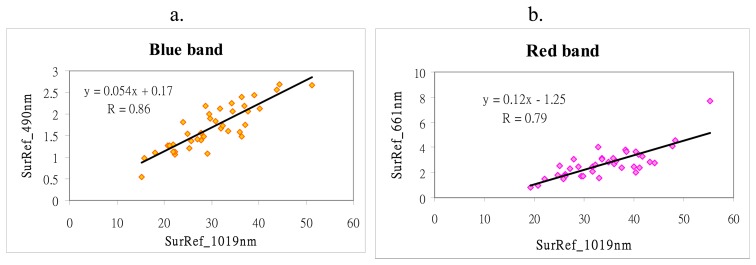
Relationship between L_surf_ at a) 490 nm and b) 661 nm, respectively, against 1,019 nm (following subtraction of the offset indicated in Figure 2) obtained from field spectra.

To test the ability to of the CHRIS 1,019 nm band to represent surface reflectance, TOA_1019_ was tested with the ground reflectance from a Cropscan MSR-16R multispectral radiometer (MSR) obtained within 30 minutes of the satellite overpass, on several dates. As a NIR band, the CHRIS 1,019 nm channel has high reflectance over vegetation, theoretically up to 50%. However Remer *et al.* [21] note that brighter surfaces than originally proposed by Kaufman *et al.* [12] ie. up to 25% are able to represent surface reflectance. While the 1,019 nm band is transparent to fine aerosols, larger aerosols scatter radiation at this wavelength, so while not totally transparent to aerosols, it is much more transparent than are the shorter visible bands. In this study the Path Reflectance (ρ_1019_) was found to be approximately 10% of TOA_1019_ ie. 3.3% (Figure 2) and this was deducted from TOA_1019_ to give L_surf1019_, before computing the empirical relationship with the visible bands. This empirical relationship was established from 42 forest ground sites using the MSR, and is given in Figure 3 for the blue (490 nm), and red (661 nm) bands. The aerosol reflectance can be derived from TOA reflectance (Equations 2-5).

To adjust the TOA_1019_ using the spectral radiometer reading, we can achieve the L_surf1019_ by:

(2) $${\text{TOA}}_{1019}-3.3\%={\text{L}}_{\text{surf}1019}$$

Equation 3 follows from the empirical relationship between surface reflectance at 1,019 and 490 nm derived from spectral radiometer (Figures 3a-c) for blue band (490 nm):

(3) $${\text{L}}_{\text{surf}490}=0.054^{*}\;{\text{L}}_{\text{surf}1019}+0.17$$

Combining 2 and 3 we derive the relationship between TOA_1019_ and L_surf490_ (Equation 4).

(4) $${\text{L}}_{\text{surf}490}=0.054^{*}\;({\text{TOA}}_{1019}-3.3\%)+0.17$$

Aerosol Reflectance at 490 nm is then derived from Equation 5 by assuming insignificant Rayleigh reflectance and a Lambertian surface:

(5) $$\rho_{490}={\text{TOA}}_{490}-\frac{T_{0}\cdot T_{S}\cdot L_{\text{surf}490}}{1-s\cdot L_{\text{surf}490}}$$

where TOA_490_ =reflectance at TOA
ρ_490_ =reflectance by Aerosol scattering
T_0_ · T_S_ =downward and upward transmittance
L_surf490_ =surface reflectance
*S* = hemispheric albedo

Finally, AOT_490_ is derived from Aerosol reflectance at 490 nm (p_490_) using the 6S radiative transfer model and an aerosol model for Hong Kong [22] and the single scattering albedo and scattering phase function were obtained from the Hong Kong AERONET station. AOT was derived at the nominal resolution of the sensor (18 m) for convenience purposes, since over a homogeneous surface such as DDV negligible adjacency effects (which would require degradation of the resolution) would be expected. AOT images were similarly computed for the 661 nm (red) band.

### 3.3. Validation

The reliability of the image-derived AOT was tested using three methods:

(i) comparison of AOT values with sunphotometer data from the AERONET station and two handheld Microtops II sunphotometers [23] deployed in rural forest and an urban forest island at the image time. (The rural forest training areas were located in lowland areas and at least 100 m from the urban edge to avoid the adjacency effect from bright urban surfaces). Since the objective of the study is to detect detailed spatial variation, and with the AERONET site located 1.5 km beyond the image area, the AERONET values are only used as an approximate reference. AERONET values were interpolated to correspond with the CHRIS bands
(ii) comparison with four air quality stations within the image area, and
(iii) visual interpretation using a Digital Elevation Model (DEM).

## 4. Results

**Figure 4.**
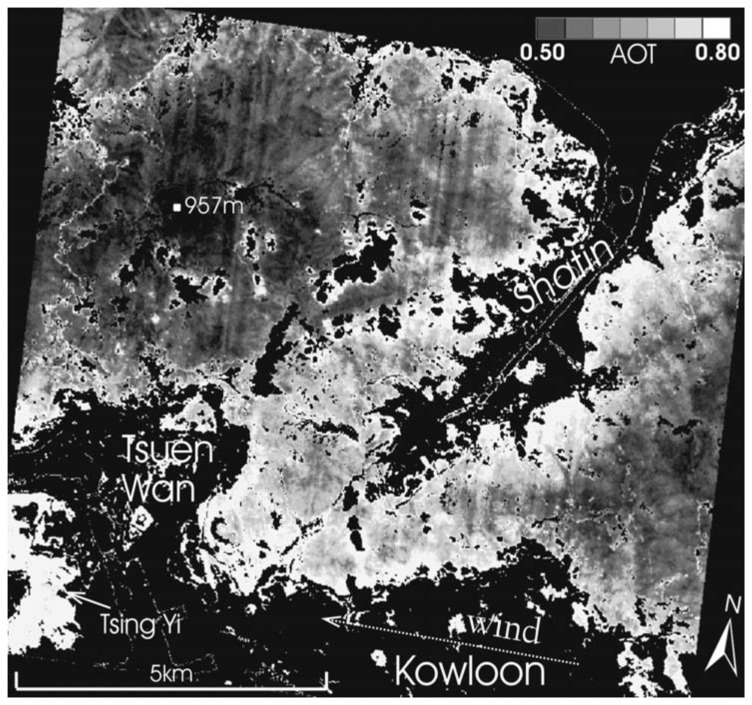
AOT image on 27-Sep-2006. Null values (black) are given for non-DDV areas.

The results suggest that L_surf490/661_ can reasonably be obtained from L_surf1019_ based on correlation coefficients of 0.86 (490 nm band) and 0.79 (661 nm band) between the ground reflectances measured by field MSR (Figure 3). This is slightly better than the R=0.75 and 0.78 obtained when the CHRIS blue and red bands respectively were tested against the Landsat SWIR band, following atmospheric corrections. Figure 4 shows the AOT image for 27-Sep-2006, derived from the 490 nm band with AOT values over rural forests and urban forest islands. AOT values are high, ranging from 0.5 to 0.8, and show significant spatial variation over the 11 × 11 km of the image. Values are highest immediately surrounding the urban areas, which also correspond to low elevations (as discussed below).

### 4.1. Comparison with ground sunphotometers

**Figure 5.**
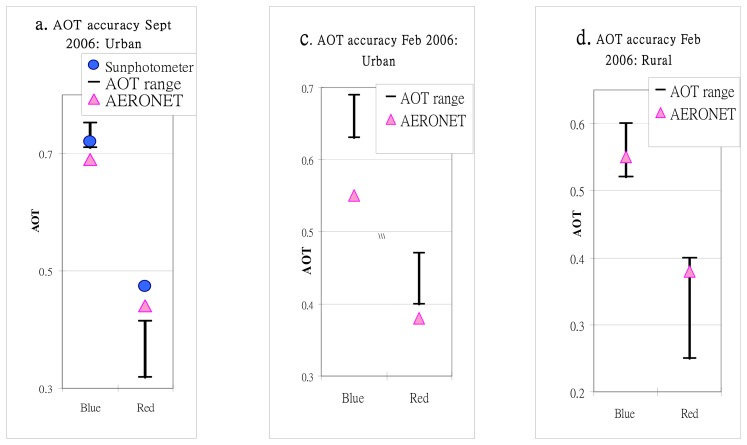
Image AOT on 27-Sep-2006. a) Urban, and b) Rural, and on 7-Feb-2006, c) Urban and d) Rural, compared with AERONET and Microtops II sunphotometer data. AOT range represents 1 s.d.

AERONET is located approximately 1.5 km south of the image area, and represents an urban site. For rural DDV areas the blue band showed the best results, with both AERONET and Microtops II values falling within the SD range of AOT values for both image dates on which field data were available (Table 1), (Figures 5b and d). For urban DDV (forest islands) (Figures 5a and c) the Microtops II reading, which unlike AERONET, was taken within the forest island, also lies within the range of image AOT (Figure 5a) but toward the lower end. For the urban forest islands, image AOT over-estimates AERONET on both dates, which may be expected since these small patches of forest of less than 200 m diameter may be affected by adjacency effects due to scattering from brighter urban pixels. This means their use for interpolation of AOT values from rural DDV areas over the urban area would be unreliable. The greater accuracy of the blue band may be explained by the higher signal-to-noise (S/N) ratio where the signal (aerosol) is higher and the surface reflectance (noise) is significantly lower, thus reducing the magnitude of uncertainty of surface reflectance. For example the S/N ratio for the blue and red bands respectively for the September-06 image (Table 2) are 5.4 (9.7/1.8) and 1.4 (3.8/2.8).

### 4.2. Comparison with ground air pollution data

Four of the EPD's air quality stations fall within the image area, and three of these are within 500 m of DDV areas (Figure 1). Although several pollutants are recorded hourly, this does not include PM2.5, and the PM10 data is not expected to show a high correlation with image AOT because of the non-selective scattering action of PM10 particles which are larger than the wavelengths used. Therefore image AOT values were compared with aerosol precursor gases (ie. the ‘urban continental’ aerosol type is formed from unstable nitrate and sulphate compounds).

**Figure 6.**
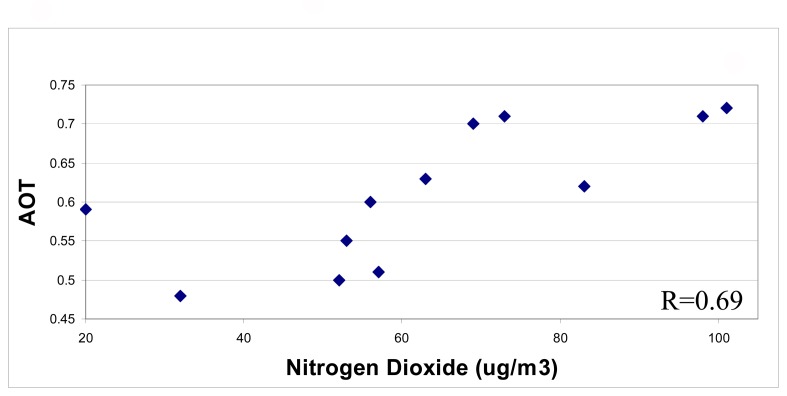
Relationship between AOT and NO_2_ from the four air quality stations within one hour of image time for the three image dates.

Over the three image dates for four stations, correlation coefficients of 0.69, 0.40 and 0.31 were obtained for NO_2_ (Figure 6), NO_x_ and SO_2_ respectively compared with image AOT_490nm_. The image time at mid-morning following peak traffic density would explain the substantial NO_2_ levels observed, as well as their correlation with fine aerosol levels derived from the image.

### 4.3. Visual interpretations

On the image date in Figure 4, 27-Sept-2006, urban NO_2_ levels are all more than four times higher than rural levels given by the Tap Mun station (5 on Figure 1). This suggests significant variation in air quality over Hong Kong at the image time, which confirms the significant spatial variation in image AOT with highest levels adjacent to urban areas (Figure 4). The figure also indicates declining air quality westwards, consonant with an east wind carrying urban pollutants from Tsuen Wan and Kowloon over the DDV areas in the west of the image, and high AOT levels over the island of Tsing Yi (bottom left of image) approach 0.8.

**Figure 7.**
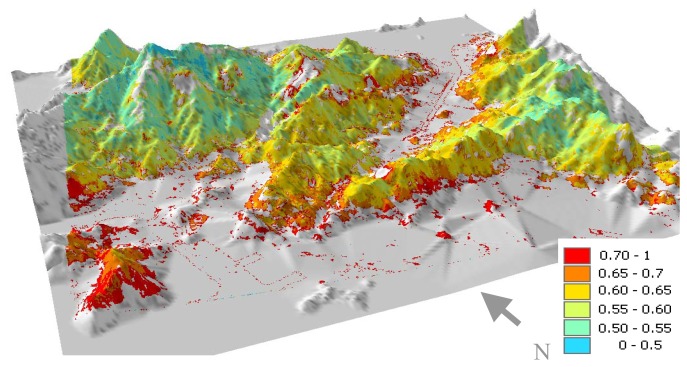
Classified AOT image draped over DEM of CHRIS image area looking NNE, from the flat, urbanized Kowloon Peninsula over the mountainous New Territories, with the small urban centres of Shatin at top right and Tsuen Wan at centre left. NB. Urban areas have no AOT values

**Figure 8.**
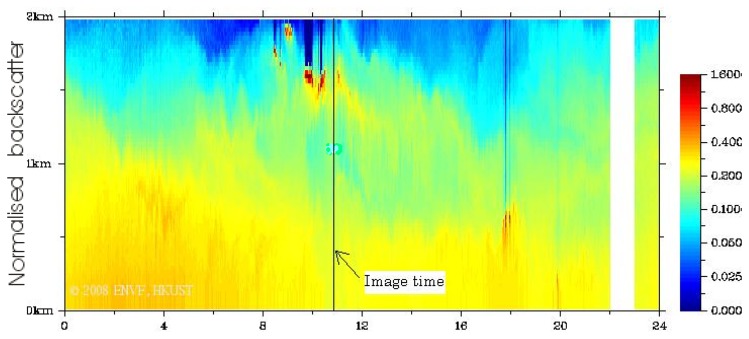
Lidar backscattering profile over 24 hours on 27-Sep-2006 (image acquired from the Institute for the Environment, the Hong Kong University of Science and Technology).

Visual interpretation is further facilitated by terrain modeling of AOT (Figure 7). Both north and south-facing slopes adjacent to urban areas are brighter (red colour denoting high AOT), indicating that the influence of surface reflectance (which would normally be higher on sunny south-facing slopes) has been successfully eliminated by the algorithm which removes the influence of L_surf_ from TOA. The terrain model also illustrates lower AOT values with higher altitude on the mountain tops between 800 and 900 m. This would be expected as a function of altitude as a local Lidar profile (Figure 8) indicted that aerosols were concentrated in the lowest 800 m at the image time, suggesting a local urban source from vehicle emissions. However AOT at the highest elevations is still higher (AOT_490nm_>0.50) than global background levels, suggesting some long-distance sources. The Lidar shows a secondary aerosol peak at ca. 1600 m height, suggesting long distance aerosol sources, which supports the observed AOT vertical distribution in the image. These three observations (Higher AOT at lower elevations, adjacent to urban areas and from east to west) were found on all three image dates.

## 5. Discussion

This study suggests that good results for air quality monitoring can be obtained from a small satellite, CHRIS/PROBA, with image-derived AOT showing considerable variation over short distances and the ability to indicate likely pollution sources more empirically than model data. The AOT product can also be used for calibrating or verifying air quality models over spatially complex regions, although degradation of the resolution may be desirable. Indeed strong relationships have been observed between satellite AOT observations from MODIS MOD04 product and PM2.5 data from ground stations, even at the coarse resolution of 10 km resolution [24-25]. In the current study the greatest error sources in AOT retrieval are thought to be due to:

(i) the assumption of linearity when converting TOA_1019_ to L_surf1019_ by deduction of the difference between them as observed by field radiometer (Figure 2). The deduction of approximately 10% of TOA_1019_ ie. 3.3% to reduce TOA_1019_ to L_surf1019_ appears effective since it is likely that the 10% relationship is linear for the larger particle sizes to which the 10% difference applies. An estimate of the error can be given, thus an error in L_surf1019_ of 1 s.d. ie. 1.46% converted to AOT would produce an error in L_surf490_ of 0.06% and in L_surf661_ of 0.16%, thus an AOT error of 0.06 in blue, and 0.04 in red. This is comparable with MODIS AOT retrievals where the error in L_surf_ due to the assumption of surface reflectance for the SWIR 2,100 nm band is given by Kaufman *et al.* [26] as +/-0.6% for blue and red bands, translating to an error in AOT of +/-0.06. Thus the error in the derivation of L_surf_ in this project is reasonable
(ii) variability in the relationship between CHRIS L_surf1019_ and L_surf_ for blue and red bands, with correlation coefficients of 0.86 and 0.79 respectively. However these compare reasonably with the 0.75 and 0.93, observed for MODIS 2,100 nm band correlated with the blue and red bands [15]
(iii) assumptions in the aerosol model used which Chu *et al.* [27] suggested can range from 0-20%, but since an aerosol model devised for Hong Kong was used, this error is likely to be considerably reduced
(iv) interpolation between AERONET and CHRIS bands which may account for 0-10% depending on the aerosol type [21].

The present study observed higher accuracies for the blue than for the red band which is similar to the findings of Chu *et al.* [27] for MODIS where the red band shows a greater effect of the error in L_surf_ due to relatively lower aerosol reflectance. The tendency for image AOT to overestimate actual AOT in the urban forest islands also supports Chu *et al.'s* [27] findings of AOT overestimation for MODIS, but in our case this may be due to the small size of the forest islands and adjacency effects from bright urban pixels. Generally the error obtained is comparable to MODIS AOT retrievals, and as with MODIS, results mainly from the uncertainty of L_surf_ which according to Remer *et al.* [15] L_surf_ derived MODIS' SWIR band varies between urban and rural, tropical and non-tropical areas, and when tested in Hong Kong this waveband gave lower accuracy for L_surf_ than t*h*e method using the CHRIS 1019nm band described here.

The study illustrates considerable variability in AOT concentrations over the image area of 11 × 11 km, which is only slightly larger than one MODIS AOT (MOD04) pixel and confirms the need for higher resolution AOT retrievals than are possible using current sensors and algorithms. The most promising technique for obtaining surface reflectance and thus aerosols over bright urban surfaces may be the Minimum Reflectance Technique (MRT) [14, 22, 28-29]. This technique appears to be reliable over heterogeneous surfaces [22, 30], but unfortunately cannot currently be developed at a resolution higher than the 500 m of MODIS, since it requires a minimum reflectance image comprised of darkest pixels from many different dates, and there is no detailed satellite sensor with adequate temporal resolution.

## 6. Conclusions

The MODIS DDV algorithm was devised for continuous global monitoring, where L_surf_ must be obtained from the image itself due to unpredictable changes in land surface characteristics. For a small satellite sensor such as CHRIS/PROBA whose radiometric calibration is not ensured, the establishment of the empirical relationship using ‘in-situ’ reflectance spectra, as used here, is desirable. For any local study area, an alternative would be to establish a local database of geo-referenced DDV sites where surface reflectance in the visible wavebands is measured in the field for different times of year.

AOT estimation from remote sensing platforms remains difficult, with errors of 5-20% given for the MODIS 10 km product MOD04, where the error in retrieval is reduced by increasing the signal-to-noise ratio by resampling to 10 km resolution. With finer resolution sensors for more spatially detailed estimates, as well as for retrieval of AOT over bright urban surfaces, the task is much more challenging, and probably requires customized platform-sensor combinations such as could be offered by small satellites if suitable AOT retrieval algorithms can be demonstrated.

## Figures and Tables

**Table 1.** AOT values from AERONET, Microtops II, and range of image AOT values for urban and rural training areas. Range represents 1 s.d.

**Urban Forest**	**Band 2: 490 nm**			**Band 7: 661 nm**		

	Dec-05	Feb-06	Sept-06	Dec-05	Feb-06	Sept-06

AERONET	-	0.55	0.69	-	0.38	0.44
Microtops II	-	-	0.72	-	-	0.47
Image AOT	0.50-0.62	0.63-0.69	0.71-0.75	0.24-0.28	0.4-0.47	0.32-0.41

**Rural Forest**	**Band 2: 490 nm**			**Band 7: 661 nm**		

	Dec-05	Feb-06	Sept-06	Dec-05	Feb-06	Sept-06

AERONET	-	0.55	0.69	-	0.38	0.44
Microtops II	-	-	0.67	-	-	0.46
Image AOT	0.43-0.50	0.52-0.6	0.63-0.69	0.21-0.26	0.25-0.4	0.24-0.39

**Table 2.** Image reflectance (%): (TOA), Surface reflectance (L_surf_) and Path (aerosol) reflectance for CHRIS 490 nm, 661 nm and 1,019 nm bands (mean values from training areas). MSR values were recorded between October and December 2006.

	**TOA**			**L_surf_**			**Path Reflectance**			**MSR (mean)**
	Dec-05	Feb -06	Sep -06	Dec-05	Feb -06	Sep -06	Dec-05	Feb -06	Sep -06	
B2 490nm	10.4	11.3	11.5	1.4	1.5	2.0	9.0	9.8	9.3	1.64
B18 1019nm	28	30.8	31.6							26.5

## References

[1] Tanré D., Deschamps P.Y., Devaux C., Herman M. (1988). Estimation of Saharan aerosol optical thickness from blurring effects in Thematic Mapper data. J. Geophys. Res..

[2] Sifakis N.I., Soulakellis N.A., Paronis D.K. (1998). Quantitative mapping of air pollution density using earth observations: a new processing method and application to an urban area. IEEE Trans. Geosci. Remote Sens..

[3] Kaufman Y., Tanré D. (1998). Algorithm for remote sensing of tropospheric aerosol from MODIS..

[4] Hsu N.C., Tsay S.C., King M.D., Herman J.R. (2004). Aerosol Properties Over Bright Reflecting Source Regions. IEEE Trans. Geosci. Remote Sens..

[5] Li Z.Q., Niu F., Lee K.H., Xin J.Y., Hao W.M., Nordgren B., Wang Y.S., Wang P.C. (2007). Validation and understanding of Moderate Resolution Imaging Spectroradiometer aerosol products (C5) using ground-based measurements from the handheld Sun photometer network in China. J. Geophys. Res..

[6] Sifakis N.I., Deschamps P.-Y. (1992). Mapping of air pollution using satellite data. Photo. Engin. Remote Sens..

[7] Retalis A., Cartalis C., Athanassiou E. (1999). Assesment of the distribution of aerosols in the area of Athens with the use of Landsat Thematic Mapper data. Int. J. Remote Sens..

[8] (2008). EPD, Past API Record.

[9] Lo J.C.F., Lau A., Fung J.C.H., Chen F. (2006). Investigation of enhanced cross-city transport and trapping of air pollutants by coastal and urban land-sea breeze circulations. J. Geophys. Res..

[10] Yuan Z., Lau A., Zhang H., Yu J.Z., Louie P.K., Fung J. (2006). Identification and spatiotemporal variations of dominant PM10 sources over Hong Kong. Atmos. Environ..

[11] Civic Exchange (2007). Relative Significance of Local vs. Regional Sources: Hong Kong's Air Pollution.

[12] Kaufman Y.J., Tanré L.A., Remer L.A., Vermote E., Chu A., Holben B.N. (1997a). Operational remote sensing of tropospheric aerosol over land from EOS moderate resolution imaging spectroradiometer. J. Geophys. Res..

[13] Levy C., Remer L.A., Martins J.V., Kaufman Y.J., Plana-fattori A., Redemann J., Wenny B. (2004). Evaluation of the MODIS aerosol retrievals over ocean and land during CLAMS. J. Atmos. Sci..

[14] Lee K.H., Kim Y.J., Hoyningen-Huene W.V., Burrow J.P. (2006). Influence of land surface effects on MODIS aerosol retrieval using the BAER method over Korea. Int. J. Remote Sens..

[15] Remer L., Tanré D., Kaufman Y. (2006). Algorithm for remote sensing of tropospheric aerosol from MODIS..

[16] ESA (1999). Exploitation of CHRIS data from the PROBA mission for science and applications..

[17] Shaker A., Nichol J.E., Wong M.S. (2008). Topographic mapping from small satellites: a case study of CHRIS/PROBA data. Photogram. Record.

[18] Garcia J.C., Moreno J. (2004). Removal of noises in CHRIS/PROBA images: application to the SPARC campaign data. http://earth.esa.int/workshops/chris_proba_04/papers/9_GARCIA.pdf.

[19] Guanter L., Alonso L., Moreno J. (2005). A method for the surface reflectance retrieval from PROBA/CHRIS data over land: application to ESA SPARC campaigns. IEEE Trans. Geosci. Remote Sens..

[20] Kaufman Y.J., Tanré D. (1992). Atmospherically resistant vegetation index (ARVI) for EOS-MODIS. IEEE Trans. Geosci. Remote Sens..

[21] Remer L.A., Kaufman Y.J., Tanré D., Mattoo S., Chu D.A., Martins J.V.m, Li R-R., Ichoku C., Levy R.C., Kleidman R.G., Eck T.F., Vermote E., Holben B.N. (2005). The MODIS aerosol algorithm, products and validation. J. Atmos. Sci..

[22] Wong M.S., Lee K.H., Nichol J.E., Li Z.Q. Retrieval of aerosol optical thickness using MODIS 500 × 500m^2^, a study in Hong Kong and Pearl River Delta region.

[23] Ichoku C., Levy R., Kaufman Y.J., Remer L.A., Li R-R., Martins V.J., Holben B.N., Abuhassan N., Slutsker I., Eck T.F., Pietras C. (2002). Analysis of the performance characteristics of the five-channel Microtops II sunphotometer for measuring aerosol optical thickness and precipitable water vapour. J. Geophys. Res..

[24] Wang J., Christopher S.A. (2003). Intercomparison between satellite-derived aerosol optical thickness and PM2.5 mass: Implications for air quality studies. Geophys. Res. Lett..

[25] Engel-Cox J.A., Holloman C.H., Coutant B.W., Hoff R.M. (2004). Qualitative and quantitative evaluation of MODIS satellite sensor data for regional and urban scale air quality. Atmos. Environ..

[26] Kaufman Y.J., Wald A.E., Remer L.A., Gao B.C., Li R.R., Flynn L. (1997b). The MODIS 2.1um channel - correlation with visible reflectance for use in remote sensing of aerosol. IEEE Trans. Geosci. Remote Sens.

[27] Chu A., Kaufman Y.J., Ichoku C., Remer L.A., Tanré D., Holben B.N. (2002). Validation of MODIS aerosol optical depth retrieval over land. Geophys. Res. Lett..

[28] Herman J.R., Celarier E.A. (1997). Earth surface reflectivity climatology at 340–380 nm from TOMS data. J. Geophys. Res..

[29] Koelemeijer R.B.A., de Haan J.F., Stammes P. (2003). A database of spectral surface reflectivity in the range 335–772 nm derived from 5.5 years of GOME observations. J. Geophys. Res..

[30] Wong M.S., Nichol J.E. A new algorithm for retrieving aerosol optical thickness over Hong Kong from MODIS satellite images. J. Geograph. Inform. Sci..

